# Systematic Review and Meta-Analysis of Extended-Spectrum Beta-Lactamase-Producing (ESBL) *Escherichia coli* in Food-Producing Animals and Animal Products in Nigeria

**DOI:** 10.3390/antibiotics15050432

**Published:** 2026-04-25

**Authors:** Yusuf Yakubu, Mohammed Sani Gaddafi, Ummukulthum Lawal Hassan, Ibrahim Idris, Laura Felicioli, Kelvin Olutimilehin Jolayemi

**Affiliations:** 1 Department of Public Health and Preventive Medicine, School of Medicine, St. George’s University, True Blue, St. George’s P.O. Box 1851, Grenada; lfelicio@sgu.edu; 2 Department of Public Health, Ministry of Animal Health, Husbandry and Fisheries, Birnin-Kebbi 860101, Kebbi State, Nigeria; 3 Department of Public Health and Preventive Medicine, Faculty of Veterinary Medicine, Usmanu Danfodiyo University, Sokoto 840103, Sokoto State, Nigeria; 4 Department of Veterinary Services, Ministry of Livestock and Fisheries Development, Sokoto 840103, Sokoto State, Nigeria; ummavet@gmail.com; 5 School of Veterinary Medicine, Texas Tech University, Amarillo, TX 79106, USA; ibidris@ttu.edu; 6 Department of Veterinary Pharmacology and Toxicology, Ahmadu Bello University, Zaria 810211, Kaduna State, Nigeria; jolayemikelvin@yahoo.com

**Keywords:** extended-spectrum beta-lactamase, *Escherichia coli*, antimicrobial resistance, food-producing animals, animal-derived food products, livestock, One Health, Nigeria

## Abstract

**Background/Objectives**: Extended-spectrum beta-lactamase (ESBL)-producing *Escherichia coli* are priority antimicrobial-resistant pathogens with significant implications for food safety and public health. Food-producing animals and their derived products represent a key interface for zoonotic transmission, yet prevalence data across Nigeria remain fragmented and unsynthesized. This systematic review and meta-analysis assessed the prevalence, species distribution, geographical patterns, and detection methods of ESBL-producing *E. coli* in food-producing animals and animal-derived food products across Nigeria. **Methods**: A comprehensive search of PubMed, Web of Science, Scopus, and African journals online was conducted for studies published between January 2000 and January 2026, following PRISMA 2020 guidelines. Twenty eligible studies collectively analyzed 5104 samples, and 984 ESBL-positive isolates were included in the meta-analysis. **Results**: The overall pooled prevalence of ESBL-producing *E. coli* was 17.0% (95% CI: 13.0–21.0%; I^2^ = 89.4%). Subgroup analysis by animal species revealed the highest pooled prevalence among caprine (32.0%, 95% CI: 17.0–52.0%), bovine (24.0%, 95% CI: 17.0–33.0%), porcine (17.0%, 95% CI: 7.0–36.0%) and avian species (13.0%, 95% CI: 9.0–19.0%). Animal-derived food products showed a pooled prevalence of 19.0% (95% CI: 17.0–21.0%). Regional analysis showed the highest pooled prevalence in South-West (19.0%, 95% CI: 13.0–27.0%) and South-South (19.0%, 95% CI: 9.0–34.0%). Studies using combined culture and molecular methods reported higher pooled prevalence (19.0%, 95% CI: 14.0–25.0%) than culture alone (12.0%, 95% CI: 8.0–18.0%). However, the difference between subgroups was not statistically significant (test for subgroup differences: *p* = 0.0563). **Conclusions**: These findings confirm extensive ESBL-producing *E. coli* circulation in Nigerian food-producing animals and highlight critical gaps in antimicrobial stewardship, veterinary surveillance, and food safety infrastructure, underscoring the urgent need for coordinated One Health strategies to contain the spread of resistant strains through the food chain.

## 1. Introduction

In the 21st century, antimicrobial resistance (AMR) has emerged as a significant challenge to the health of humans and animals, affecting public health, food safety, and food security globally [1]. The transmission of AMR between humans and animals is widely recognized as a critical component of this challenge, driven largely by the excessive and indiscriminate use of antibiotics across human medicine, veterinary practice, and agriculture [2,3]. Consequently, antibiotic resistance contributes directly to treatment failure, compromising animal health, welfare, and productivity [4,5].

Among the most clinically important resistant pathogens are extended-spectrum β-lactamase (ESBL)-producing *Escherichia coli*, which have been classified by the World Health Organization as critical priority pathogens [6]. ESBL enzymes confer resistance to a broad spectrum of β-lactam antibiotics, including penicillins and third-generation cephalosporins, thereby limiting therapeutic options [6]. Although initially associated with nosocomial infections, ESBL-producing *E. coli* are now increasingly detected in a wide range of animal species, particularly food-producing animals and their derived products [7,8].

This epidemiological shift highlights the expanding role of livestock production systems as key reservoirs and transmission pathways for ESBL-producing *E. coli*. The widespread use of antibiotics in animal husbandry for growth promotion, disease prevention, and treatment, often without veterinary supervision, creates sustained selective pressure for resistant bacterial populations [9]. These resistant organisms can subsequently spread through the food chain, direct animal contact, and environmental pathways [10]. Food-producing animals therefore act as important reservoirs of ESBL-producing *E. coli*, as documented globally across multiple livestock species, including poultry, cattle, and pigs [11,12,13,14,15].

Of particular concern is the contamination of animal-derived food products such as meat, milk, and eggs, which serve as direct vehicles for transmitting ESBL-producing *E. coli* to human consumers [8]. This elevates ESBL contamination from a purely veterinary issue to a major food safety and public health concern.

The burden of AMR is particularly severe in low- and middle-income countries (LMICs), where surveillance systems and regulatory enforcement for antimicrobial use are often limited [16]. In Nigeria, rapid population growth and increasing demand for animal protein have driven expansion in livestock production systems, including poultry, cattle, and pig farming [17]. However, improvements in biosecurity and antimicrobial stewardship have not kept pace with this expansion. Critically important antibiotics, including third-generation cephalosporins, remain widely accessible over the counter and are frequently used without veterinary prescription [18].

As a result, multiple studies have reported the presence of ESBL-producing *E. coli* in animals, animal-derived food products, and farm environments across Nigeria [19]. However, these data remain fragmented, with substantial variation in study design, geographic coverage, animal species, and diagnostic methodologies. Importantly, no previous systematic review has comprehensively compiled ESBL-producing *E. coli* prevalence across Nigeria’s geopolitical zones, limiting the ability to identify regional patterns and inform targeted interventions.

Addressing this gap requires a One Health approach that recognizes the interconnectedness of human, animal, and environmental health. ESBL-producing *E. coli* can move across these interfaces through foodborne transmission, environmental contamination, and direct contact [10,19]. In Nigeria, where food hygiene practices and cold-chain infrastructure remain inconsistent, contamination of animal-derived food products represents a significant pathway for human exposure [20]. Despite this, surveillance efforts remain disproportionately focused on live animals, with limited attention to food products reaching consumers.

Therefore, this study aimed to systematically review and conduct a meta-analysis of ESBL-producing *Escherichia coli* in food-producing animals and animal-derived food products in Nigeria. Specifically, the study sought to estimate pooled prevalence, evaluate species-specific and regional distribution patterns, and identify critical gaps in research and surveillance to inform evidence-based One Health interventions.

## 2. Results

### 2.1. Results of Search

**Figure 1:**
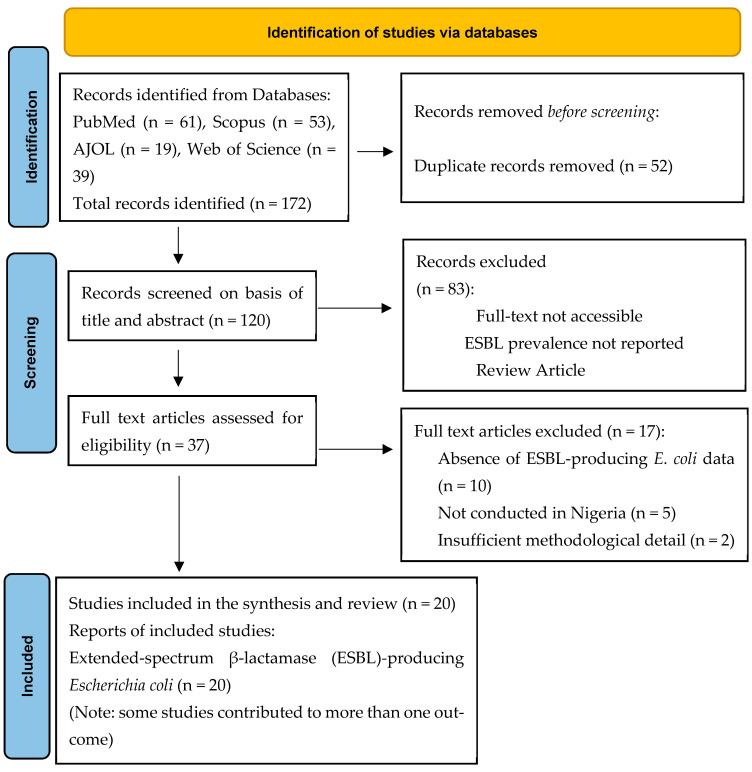
PRISMA flow chart (Source: Page MJ et al., 2021 [24] or http://www.prisma-statement.org/ (accessed on 15 January 2026).

A systematic search of four databases, PubMed, Scopus, Web of Science, and African Journals Online (AJOL), retrieved 172 records in total. Duplicate removal eliminated 52 records, resulting in 120 unique studies that proceeded to title and abstract screening. Of these, 83 records were excluded at the screening stage for failing to meet the predefined eligibility criteria, and 37 full-text articles advanced to detailed evaluation. Upon full-text review, 17 articles were excluded for the following reasons: conducted outside Nigeria (n = 10), no reportable ESBL-producing *E. coli* data (n = 5), and inadequate methodological detail to permit quality assessment (n = 2). The remaining 20 studies satisfied all inclusion criteria and were incorporated into the final synthesis and meta-analysis (Figure 1).

### 2.2. Characteristics of Included Studies

This systematic review and meta-analysis included 20 studies published over a 25-year period spanning 2000 to 2025, focusing on the prevalence of extended-spectrum β-lactamase (ESBL)-producing *Escherichia coli* among food-producing animals and animal-derived food products in Nigeria. Across all included studies, a combined total of 5104 samples were examined, from which 984 isolates were confirmed as ESBL-producing (Table 1).

Geographically, the included studies covered several states across multiple geopolitical regions of Nigeria. At the geopolitical level, the highest was South-West (n = 7), followed by South-East (n = 4), North-Central (n = 4), North-West (n = 1), North-East (n = 2), South-South (n = 1) and South-West/North-Central (n = 1). At the state level, studies were most frequently conducted in Oyo (n = 4), followed by Plateau (n = 2) and Borno (n = 2). Single studies were conducted in Sokoto (n = 1), Anambra (n = 1), Delta (n = 1), Ebonyi (n = 1), Ekiti (n = 1), Federal Capital Territory Abuja (n = 1), Imo (n = 1), Kwara (n = 1), and Lagos (n = 1). Combined studies in states without separation of data according to location had Enugu and Ebonyi (n = 1), FCT and Lagos (n = 1), and Oyo and Osun (n = 1) (Table 1). Sample sizes varied widely across studies, ranging from 16 samples [21] to 1052 samples [22]. Several other studies analyzed large sample sizes, including 300 samples [23] and 272 samples [16] (Table 1).

Regarding animal species and sample sources, the studies investigated a broad range of food-producing animals and animal-derived food products. The most frequently studied species was avian (n = 12 studies), followed by bovine (n = 8) and porcine (n = 4), caprine (n = 2), ovine (n = 2) and food products (n = 4), with 2 studies on meat and 1 each on milk and egg. When categorized by sample type, the majority of studies investigated live animals (n = 16), while a smaller proportion examined animal-derived food products (n = 4) such as meat, milk, and eggs. With respect to laboratory detection methods, most studies employed combined phenotypic culture and molecular techniques (n = 14) for ESBL detection, while a smaller number used phenotypic culture-based methods alone (n = 6) (Table 1).

**Table 1:** Study Characteristics.

Study	Study Design	Species/Sample	Total (n)	ESBL+ (n)	State	Region	Method	Sample Type
[16]	Cross-sectional	Bovine	272	44	FCT and Lagos	North-Central/South-West	Culture and Molecular	Animal
[21]	Cross-sectional	Caprine	16	8	Oyo and Osun	South-West	Culture and Molecular	Animal
		Ovine	18	1				Animal
		Avian	18	7				Animal
		Bovine	18	6				Animal
		Porcine	19	8				Animal
		Food Product	31	7				Meat
[22]	Cross-sectional	Food Product	1052	212	Plateau	North-Central	Culture and Molecular	Milk
[23]	Cross-sectional	Avian	300	42	Enugu and Ebonyi	South-East	Culture and Molecular	Animal
[25]	Cross-sectional	Bovine	238	103	Lagos	South-West	Culture and Molecular	Animal
		Avian	210	80				Animal
		Porcine	152	28				Animal
[26]	Cross-sectional	Avian	200	18	Oyo	South-West	Culture and Molecular	Animal
[27]	Cross-sectional	Avian	181	9	Kwara	North-Central	Culture and Molecular	Animal
[28]	Cross-sectional	Avian	200	20	Oyo	South-West	Culture	Animal
		Avian	93	6				Animal
		Avian	93	3				Animal
[29]	Cross-sectional	Avian	111	13	FCT	North-Central	Culture and Molecular	Animal
[30]	Cross-sectional	Bovine	120	35	Ebonyi	South-East	Culture and Molecular	Animal
[31]	Cross-sectional	Avian	94	27	Delta	South-South	Culture	Animal
		Bovine	88	10				Animal
[32]	Cross-sectional	Bovine	250	53	Oyo	South-West	Culture and Molecular	Animal
		Avian	24	4				Animal
[33]	Cross-sectional	Avian	100	15	Oyo	South-West	Culture and Molecular	Animal
		Porcine	100	3				Animal
[34]	Cross-sectional	Avian	165	10	Sokoto	North-West	Culture and Molecular	Animal
[35]	Cross-sectional	Avian	96	21	Borno	North-East	Culture	Animal
[36]	Cross-sectional	Caprine	76	18	Borno	North-East	Culture	Animal
		Ovine	42	2				Animal
[37]	Cross-sectional	Food Product	47	8	Imo	South-East	Culture and Molecular	Meat
[38]	Cross-sectional	Food Product	270	42	Plateau	North-Central	Culture	Egg
[39]	Cross-sectional	Bovine	200	79	Ekiti	South-West	Culture and Molecular	Animal
		Porcine	150	35				Animal
[40]	Cross-sectional	Bovine	60	7	Anambra	South-East	Culture	Animal

Studies reporting multiple subgroups (e.g., different animal species, sample types, or diagnostic methods) are presented as separate entries to reflect subgroup-specific data included in the meta-analysis.

### 2.3. Risk of Bias and Assessment of Study Quality

The methodological rigor of all 20 included studies was evaluated through the application of the Joanna Briggs Institute (JBI) critical appraisal checklist for prevalence studies. In accordance with the predefined scoring criteria, the predominant proportion of studies, which was 14 (70.0%), fulfilled the threshold for high methodological quality (scores ≥ 70%). Two studies (10.0%) were adjudged to be of moderate quality (scores 50–69%), while the remaining four studies (20.0%) were deemed to be of low methodological quality (scores < 50%). Studies attaining high-quality designations were characteristically distinguished by clearly delineated sampling frameworks, statistically adequate sample sizes, comprehensive methodological documentation, and the consistent application of internationally recognized laboratory protocols for the detection and confirmation of ESBL-producing *Escherichia coli*. Studies categorized as moderate quality typically lacked explicit descriptions of sampling strategies or did not provide clear justification for sample size determination. In contrast, studies classified as low quality often relied on convenience sampling or provided limited methodological detail regarding laboratory procedures or study design.

**Figure 2:**
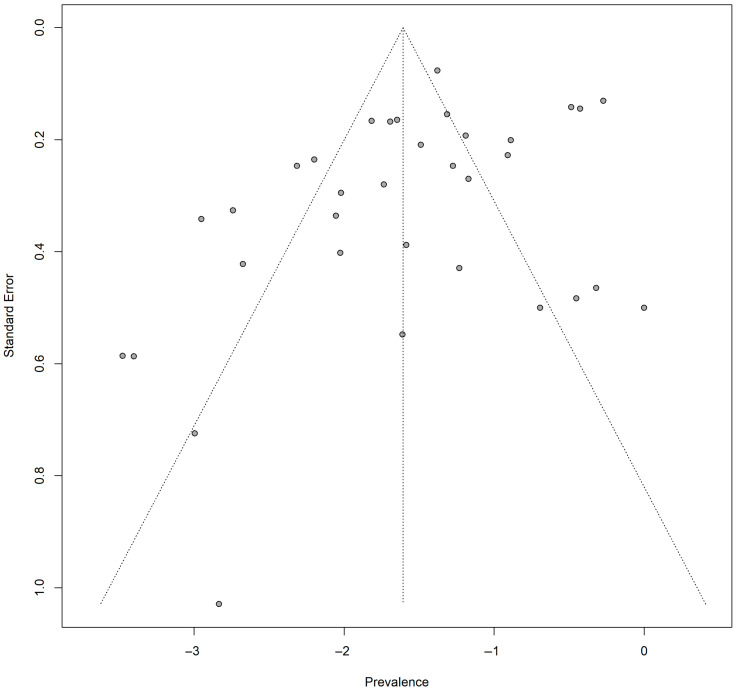
Funnel plot assessing publication bias for studies reporting extended-spectrum β-lactamase (ESBL)-producing *Escherichia coli* prevalence (Egger’s t = −2.17, df = 18, *p* = 0.0375) in Nigeria.

To evaluate the robustness and reliability of the pooled prevalence estimate, a sensitivity analysis was performed through the systematic exclusion of studies adjudged to be of low methodological quality based on the JBI critical appraisal criteria. After removal of the four low-quality studies, the meta-analysis included 16 contributing datasets (k = 16). The pooled prevalence estimate remained largely unchanged compared with the primary analysis, and substantial between-study heterogeneity persisted (I^2^ = 82.6%, *p* < 0.001) (Figure 2). These findings substantiate that the overall pooled prevalence estimate was not disproportionately influenced by studies with an elevated risk of bias, thereby affirming the stability and methodological robustness of the meta-analytic results.

Visual inspection of the funnel plot suggested slight asymmetry. Egger’s regression test revealed statistically significant asymmetry in the funnel plot (t = −2.17, df = 32, *p* = 0.0375) (Figure 2), indicative of the presence of small-study effects or potential publication bias within the included literature. However, given the substantial between-study heterogeneity observed in the meta-analysis, this asymmetry is likely to reflect a combination of small-study effects and true underlying heterogeneity rather than publication bias alone. Therefore, these findings should be interpreted with caution. Although trim-and-fill methods can be used to explore potential publication bias, their reliability is limited in the presence of high heterogeneity, and therefore such adjustments were not applied in this study.

### 2.4. Overall Pooled Prevalence of ESBL-Producing Escherichia coli

**Figure 3:**
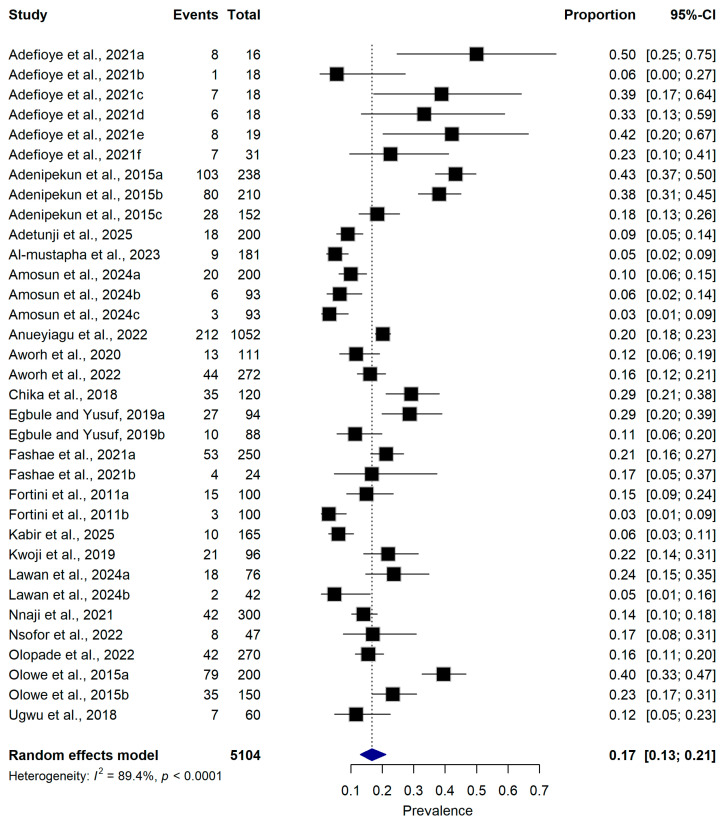
Forest plot illustrating the pooled prevalence of extended-spectrum β-lactamase (ESBL)-producing *Escherichia coli* across 20 studies ([16,21,22,23,25,26,27,28,29,30,31,32,33,34,35,36,37,38,39,40]) conducted in Nigeria. The pooled estimate was generated employing a random-effects meta-analysis model. The reported proportion reflects the prevalence of ESBL-producing *E. coli* identified in samples derived from food-producing animals and animal-derived food products, utilizing either culture-based or molecular diagnostic methodologies. Individual study estimates are denoted by squares, the dimensions of which are proportional to their respective inverse variance weights, while horizontal lines delineate the 95% confidence intervals (CI). The pooled prevalence estimate is represented by the diamond symbol. The I^2^ statistic quantifies the magnitude of between-study heterogeneity. Total number of included studies: 20; aggregate sample size: 5104.

A total of 20 studies comprising 5104 samples from food-producing animals and animal-derived food products in Nigeria were included in the meta-analysis. Across these studies, 984 ESBL-producing *Escherichia coli* isolates were identified. The overall pooled prevalence of ESBL-producing *E. coli* was 17.0% (95% CI: 13.0–21.0%) under a random-effects model. Substantial between-study heterogeneity was observed (I^2^ = 89.4%, *p* < 0.0001) (Figure 3). The prevalence estimates reported by individual studies varied considerably, ranging from 3.0% (95% CI: 1.0–9.0%) to 50.0% (95% CI: 25.0–75.0%) across the included studies (Figure 3).

### 2.5. Subgroup Analysis

In order to systematically investigate potential sources underlying the observed heterogeneity, subgroup analyses were carried out stratifying studies according to animal species, sample origin, ESBL detection method, geographical region, and state.

#### 2.5.1. Animal Species

**Figure 4:**
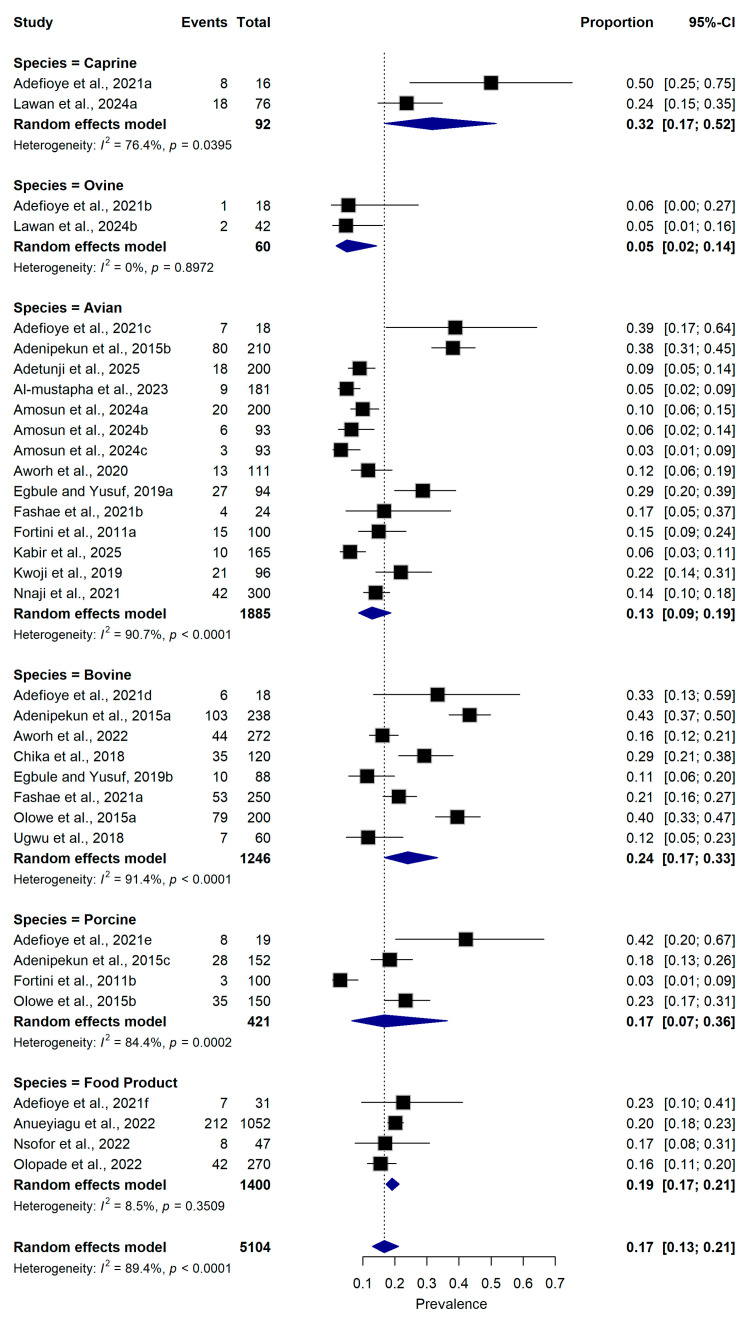
Forest plot showing subgroup analyses of ESBL-producing *Escherichia coli* prevalence stratified by animal species and animal-derived food products ([16,21,22,23,25,26,27,28,29,30,31,32,33,34,35,36,37,38,39,40]). The pooled estimate was derived using a random-effects meta-analysis model. The proportion represents the prevalence of ESBL-producing *E. coli* detected in samples collected from food-producing animals and animal-derived food products using culture-based or molecular diagnostic methods. Squares represent individual study estimates, with sizes proportional to the inverse variance weighting, while horizontal lines indicate 95% confidence intervals (CI). The diamond represents the pooled prevalence estimate. I^2^ indicates the degree of between-study heterogeneity. Total number of studies: 20; total sample size: 5104.

Subgroup analysis by animal species revealed variation in the prevalence of ESBL-producing *E. coli* among different livestock species and food products. The pooled prevalence was highest among caprine (32.0%, 95% CI: 17.0–52.0%; n = 2 datasets), followed by bovine (24.0%, 95% CI: 17.0–33.0%; n = 8 datasets). Studies involving porcine (17.0%, 95% CI: 7.0–36.0%; n = 4 datasets) and avian (13.0%, 95% CI: 9.0–19.0%; n = 14 datasets) also reported moderate prevalence estimates. Lower prevalence values were observed among ovine (5.0%, 95% CI: 2.0–14.0%; n = 2 datasets). Animal-derived food products had an aggregated prevalence of 19.0% (95% CI: 17.0–21.0%; n = 4 datasets). Considerable heterogeneity was observed among some species groups, particularly avian (I^2^ = 90.7%, *p* < 0.0001), porcine (I^2^ = 84.4%, *p* < 0.0001), and bovine (I^2^ = 91.4%, *p* < 0.0001) (Figure 4).

#### 2.5.2. Detection Method

**Figure 5:**
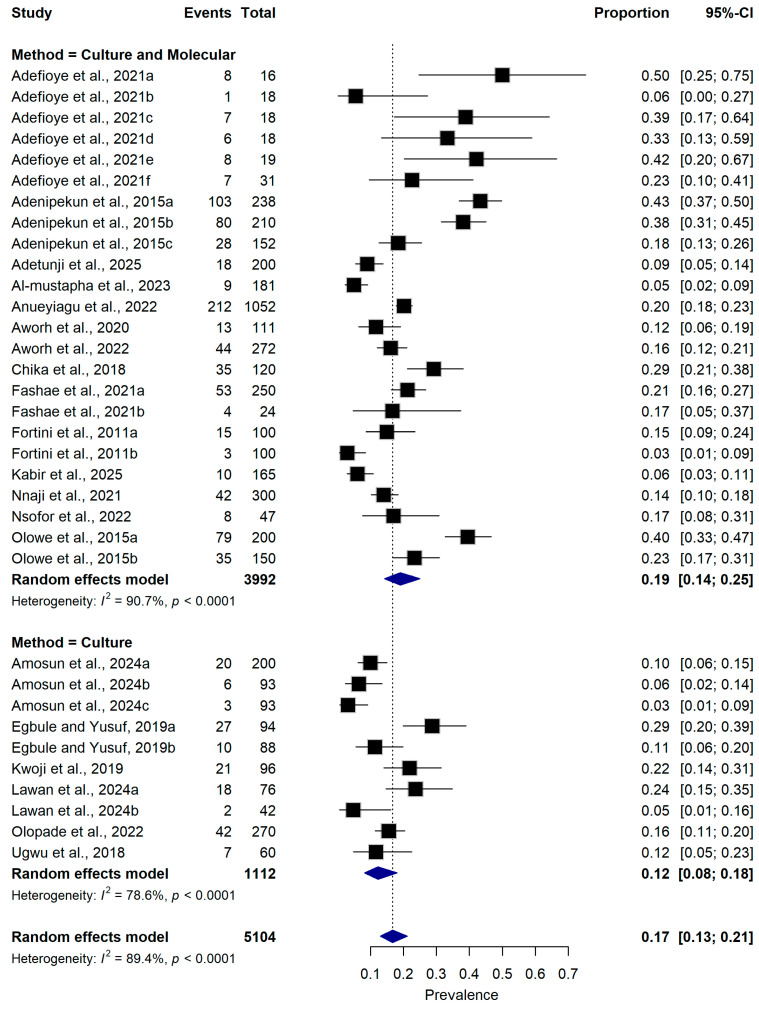
Forest plot showing subgroup analysis of ESBL-producing *Escherichia coli* prevalence according to diagnostic methods used for detection ([16,21,22,23,25,26,27,28,29,30,31,32,33,34,35,36,37,38,39,40]). The pooled estimate was derived using a random-effects meta-analysis model. The proportion represents the prevalence of ESBL-producing *E. coli* detected in samples collected from food-producing animals and animal-derived food products using culture-based or molecular diagnostic methods. Squares represent individual study estimates, with sizes proportional to the inverse variance weighting, while horizontal lines indicate 95% confidence intervals (CI). The diamond represents the pooled prevalence estimate. I^2^ indicates the degree of between-study heterogeneity. Total number of studies: 20; total sample size: 5104.

When stratified by ESBL detection method, studies using combined culture and molecular detection techniques reported a pooled prevalence of 19.0% (95% CI: 14.0–25.0%; n = 24 datasets). In contrast, studies relying on culture-based phenotypic methods alone reported a lower pooled prevalence of 12.0% (95% CI: 8.0–18.0%; n = 10 datasets). Substantial heterogeneity persisted within both methodological subgroups (I^2^ = 90.7% and 78.6%, respectively; *p* < 0.001) (Figure 5). However, the difference between subgroups was not statistically significant (test for subgroup differences: *p* = 0.0563).

#### 2.5.3. Sample Type

**Figure 6:**
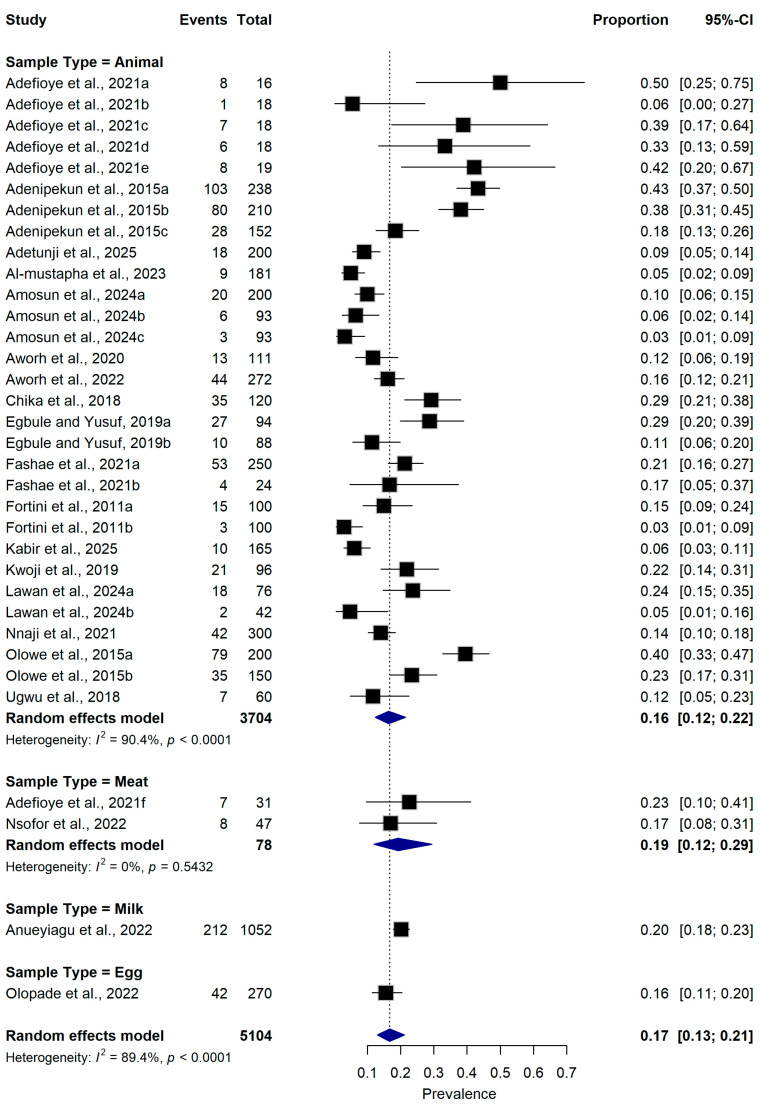
Forest plot showing subgroup analysis of ESBL-producing *Escherichia coli* prevalence according to sample types ([16,21,22,23,25,26,27,28,29,30,31,32,33,34,35,36,37,38,39,40]). The pooled estimate was derived using a random-effects meta-analysis model. The proportion represents the prevalence of ESBL-producing *E. coli* detected in samples collected from food-producing animals and animal-derived food products using culture-based or molecular diagnostic methods. Squares represent individual study estimates, with sizes proportional to the inverse variance weighting, while horizontal lines indicate 95% confidence intervals (CI). The diamond represents the pooled prevalence estimate. I^2^ indicates the degree of between-study heterogeneity. Total number of studies: 20; total sample size: 5104.

Subgroup analysis based on sample type indicated that studies involving live animals (n = 30 datasets) reported a pooled prevalence of 16.0% (95% CI: 12.0–22.0%), whereas studies examining animal-derived food products reported a pooled prevalence of 19.0% (95% CI: 12.0–29.0%) for meat, 20.0% (95% CI: 18.0–23.0%) for milk and 16.0% (95% CI: 11.0–20.0%) for egg. Heterogeneity remained substantial among studies involving animals (I^2^ = 90.4%, *p* < 0.0001) (Figure 6).

#### 2.5.4. Geographical Region

**Figure 7:**
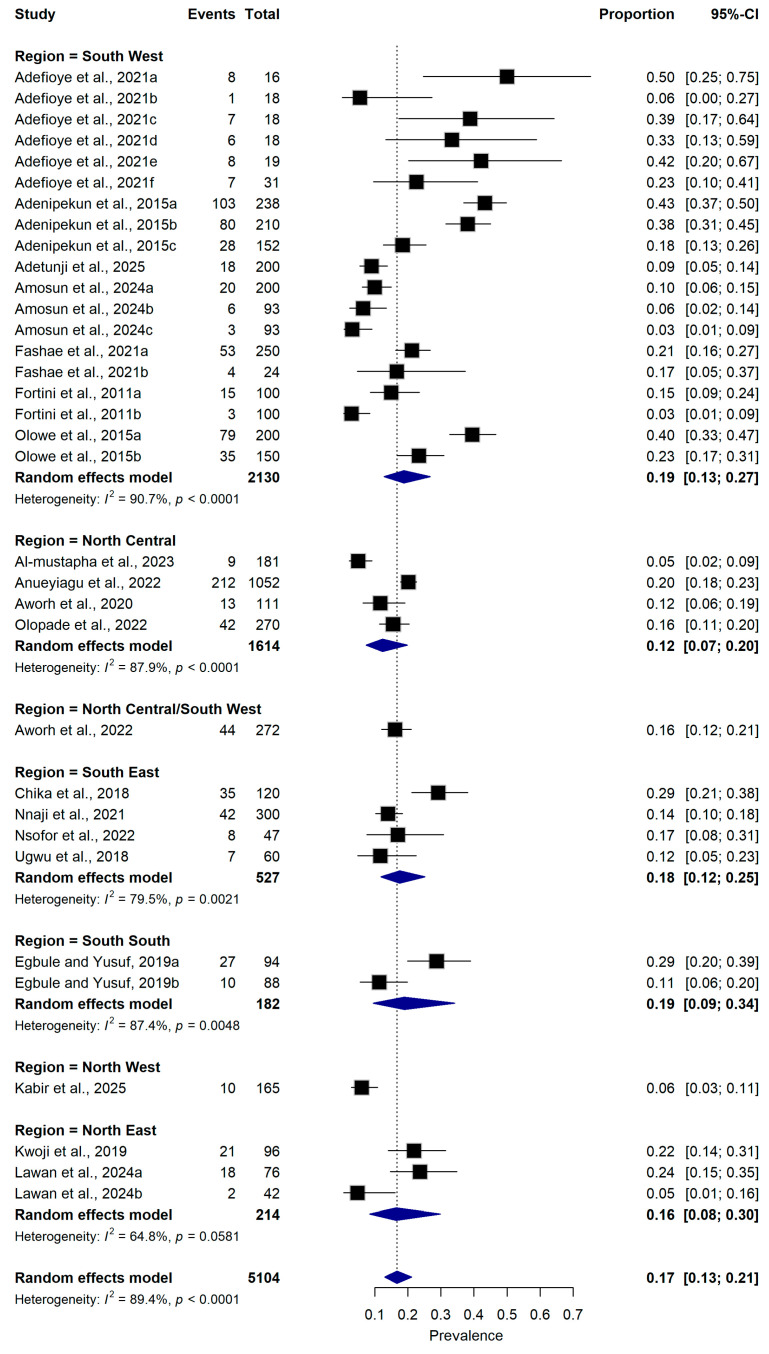
Forest plot illustrating subgroup analysis of ESBL-producing *Escherichia coli* prevalence stratified by geopolitical region in Nigeria ([16,21,22,23,25,26,27,28,29,30,31,32,33,34,35,36,37,38,39,40]). The pooled estimate was derived using a random-effects meta-analysis model. The proportion represents the prevalence of ESBL-producing *E. coli* detected in samples collected from food-producing animals and animal-derived food products using culture-based or molecular diagnostic methods. Squares represent individual study estimates, with sizes proportional to the inverse variance weighting, while horizontal lines indicate 95% confidence intervals (CI). The diamond represents the pooled prevalence estimate. I^2^ indicates the degree of between-study heterogeneity. Total number of studies: 20; total sample size: 5104.

Regional subgroup analysis demonstrated variability in prevalence across Nigeria’s geopolitical zones. The pooled prevalence was highest in the South-West (19.0%, 95% CI: 13.0–27.0%; n = 19 datasets) and South-South (19.0%, 95% CI: 9.0–34.0%; n = 2 datasets). Studies conducted in the South-East (n = 4) reported a pooled prevalence of 18.0% (95% CI: 12.0–25.0%), while those in the North-East (n = 3) reported 16.0% (95% CI: 8.0–30.0%). The North-Central (n = 4) had a pooled prevalence of 12.0% (95% CI: 7.0–20.0%), whereas North-West (n = 1) reported a prevalence estimate of 6.0% (95% CI: 3.0–11.0%). Heterogeneity remained substantial among studies in South-West (I^2^ = 90.7%, *p* < 0.0001), North-Central (I^2^ = 87.9%, *p* < 0.0001), South-East (I^2^ = 79.5%, *p* < 0.0001) (Figure 7).

#### 2.5.5. State

**Figure 8:**
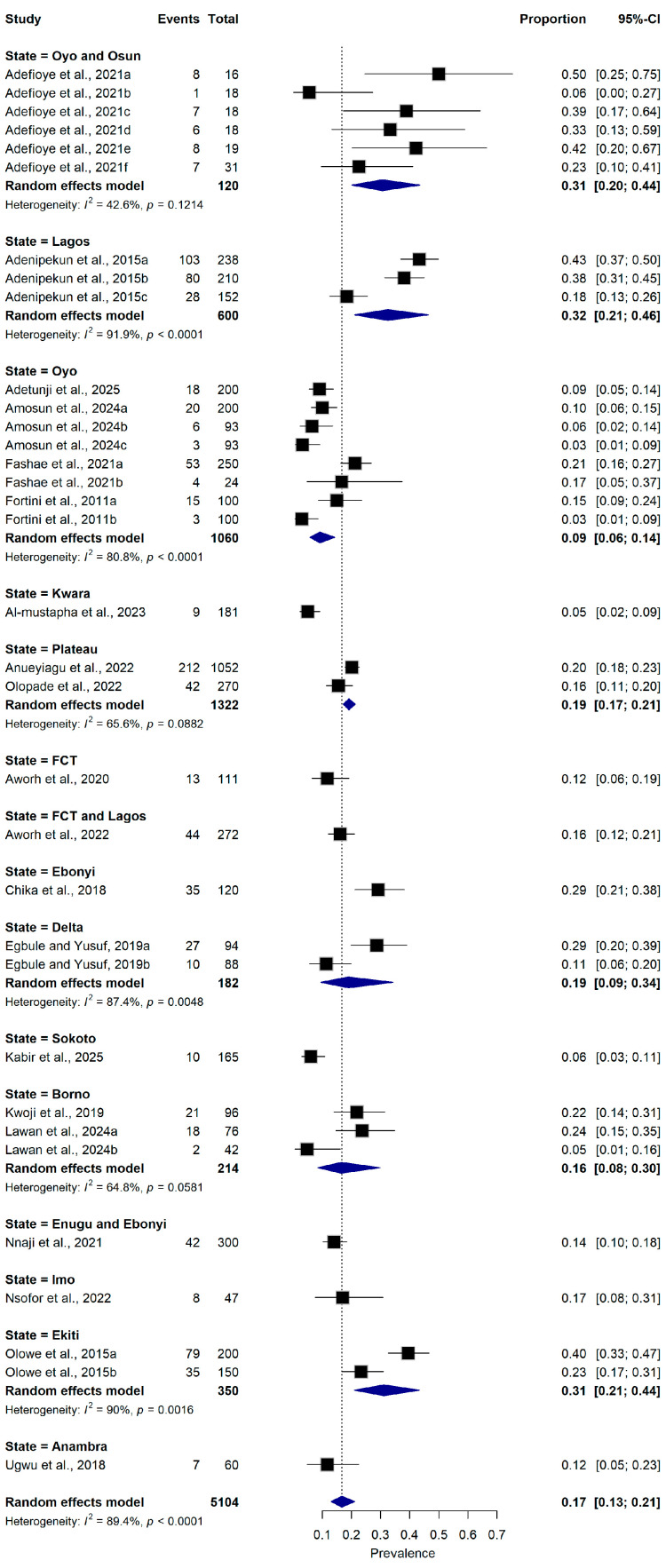
Forest plot illustrating subgroup analysis of ESBL-producing *Escherichia coli* prevalence stratified by states in Nigeria ([16,21,22,23,25,26,27,28,29,30,31,32,33,34,35,36,37,38,39,40]). The pooled estimate was derived using a random-effects meta-analysis model. The proportion represents the prevalence of ESBL-producing *E. coli* detected in samples collected from food-producing animals and animal-derived food products using culture-based or molecular diagnostic methods. Squares represent individual study estimates, with sizes proportional to the inverse variance weighting, while horizontal lines indicate 95% confidence intervals (CI). The diamond represents the pooled prevalence estimate. I^2^ indicates the degree of between-study heterogeneity. Total number of studies: 20; total sample size: 5104.

Studies conducted in Lagos (n = 3 datasets) reported a pooled prevalence of 32.0% (95% CI: 21.0–46.0%), while studies from Oyo (n = 8 datasets) reported 9.0% (95% CI: 6.0–14.0%), Plateau (n = 2 datasets) reported a pooled prevalence of 19.0% (95% CI: 17.0–21.0%), Delta (n = 2 datasets) reported a pooled prevalence of 19.0% (95% CI: 9.0–34.0%), Borno (n = 3 datasets) reported a pooled prevalence of 16.0% (95% CI: 8.0–30.0%), Ekiti (n = 2 datasets) reported a pooled prevalence of 31.0% (95% CI: 21.0–44.0%). Heterogeneity remained substantial among studies in Lagos (I^2^ = 91.9%, *p* < 0.0001), Oyo (I^2^ = 80.8%, *p* < 0.0001), and Delta (I^2^ = 87.4%, *p* < 0.0001) (Figure 8).

## 3. Discussion

To our knowledge, this is the first systematic review and meta-analysis to specifically compile evidence on ESBL-producing *Escherichia coli* across food-producing animals and animal-derived food products in Nigeria. Previous studies have either focused on human or environmental reservoirs, reported individual animal species in isolation, or lacked quantitative synthesis through meta-analysis. As a result, a comprehensive understanding of the burden and distribution of ESBL-producing *E. coli* within Nigeria’s livestock–food continuum has remained limited.

The overall pooled prevalence of 17.0% aligns closely with the broader sub-Saharan African (SSA) region data, where a meta-analysis across 196 studies reported an overall ESBL-producing *E. coli* prevalence of 20.76%, with animals as the highest-burden source at 29.15% and West Africa showing the highest sub-regional rate at 22.80% [19]. A complementary West African synthesis reported 16.8% overall prevalence, with Nigeria-specific estimates around 18.3% [41]. Nigeria’s modestly lower figure in this review likely reflects its narrower focus on livestock and derived products excluding broader human and environmental compartments while still confirming the livestock sector as a primary reservoir amid shared regional drivers such as indiscriminate antibiotic use, weak regulatory enforcement, and limited veterinary oversight [42]. In contrast to high-income settings, where targeted stewardship has reduced livestock ESBL prevalence over time [13], the Nigerian food animal sector continues to operate largely without the institutional controls necessary to contain resistance dissemination. The dominance of *bla*_CTX-M-15_ across both livestock and human clinical isolates in West Africa underscores the convergence of ESBL epidemiology across the human–animal interface, irrespective of healthcare infrastructure [13].

Situating Nigeria’s 17.0% pooled prevalence within the broader global landscape reveals important patterns that both contextualize the current findings and highlight the structural determinants of ESBL burden across livestock systems. In high-income settings, sustained regulatory reform and antimicrobial stewardship programmes have driven measurable reductions in livestock ESBL prevalence. Monitoring data from the European Union indicate that ESBL-producing *E. coli* in retail chicken meat in the Netherlands declined significantly following national reductions in antibiotic use, with ESBL-producing Enterobacteriaceae in retail poultry products falling substantially after mandatory use restrictions were introduced from 2010 onwards [43,44]. This decline demonstrates that regulatory enforcement of antimicrobial stewardship can produce measurable reductions in food chain contamination. In contrast, high burdens persist across low- and middle-income settings in Asia, Latin America, and Africa, where regulatory oversight of veterinary antimicrobial use remains limited. A One Health systematic review and meta-analysis from Bangladesh reported a pooled ESBL-producing *E. coli* prevalence of 22% in animals (95% CI: 9–34%), with *bla*_CTX-M_ variants predominant and substantial heterogeneity attributed to unregulated antibiotic use and weak veterinary oversight [45]. Studies from China have documented even higher burdens, with ESBL-producing *bla*_CTX-M_ variants detected in over 75% of food-producing animals across multiple provinces, carried predominantly on conjugative plasmids facilitating cross-species dissemination [46]. In Latin America, a systematic review of ESBL-producing *E. coli* across South America documented _CTX-M_-type enzymes as the dominant ESBL family across animal, food, and environmental reservoirs, with _CTX-M-2_ predominant in Brazil and _CTX-M-1_ in Colombia, a genetic profile that overlaps substantially with isolates circulating in African livestock and underscores the global dissemination of resistance plasmids rather than purely localized emergence [47]. Across all regions, the contamination of animal-derived food products with ESBL-producing *E. coli* at retail has been documented, with rates in LMICs generally ranging from 15 to 30%, compared with substantially lower levels in high-income countries following food safety interventions targeting slaughter hygiene and cold-chain management [8]. Nigeria’s estimated 17.0% overall prevalence and 19.0% food product prevalence therefore positions the country within the LMIC range, but closer to the lower end than high-burden settings in Asia, indicating that the situation, while serious, has not yet reached the critical levels observed in the most heavily affected food animal systems globally and that timely intervention could still meaningfully alter the trajectory.

The substantial heterogeneity observed across studies (I^2^ = 89.4%) likely reflects real differences in study design, populations, and methodological approaches rather than random variation alone. One important contributor is variation in diagnostic methods, as studies employing combined phenotypic and molecular techniques reported higher prevalence estimates than those relying solely on culture-based methods. This difference reflects the greater sensitivity of molecular approaches in detecting ESBL-associated resistance genes, including those that may not be phenotypically expressed under laboratory conditions.

Additional contributors to heterogeneity include variation across animal species driven by differences in husbandry practices, antimicrobial use intensity, and biosecurity standards, geographical differences in livestock production systems, antimicrobial access, and the inclusion of both live animals and food products, which introduces variability related to slaughter hygiene and post-production contamination.

Despite subgroup analyses, substantial heterogeneity persisted within categories, indicating that additional unmeasured factors such as farm-level practices, antimicrobial usage intensity, and study-specific methodological differences may also play important roles. Therefore, the pooled estimates should be interpreted with caution, and future studies incorporating standardized methodologies and detailed reporting are needed to better characterize these sources of variability. Although meta-regression could be used to further explore sources of heterogeneity, it was not performed in this study due to the limited number of studies within several subgroups, uneven distribution of study-level variables, and the risk of model overfitting, which may lead to unreliable estimates.

The overall certainty of evidence from this meta-analysis is best considered moderate. While most included studies were of moderate to high methodological quality, confidence in the pooled estimates is limited by the high degree of inconsistency observed across studies. This variability, together with differences in study populations, diagnostic approaches, and uneven geographical representation, reduces the precision and generalizability of the findings. In addition, although evidence of publication bias was suggested, its interpretation remains uncertain in the context of substantial heterogeneity. These considerations indicate that the findings provide a robust indication of the presence of ESBL-producing *E. coli* in Nigeria’s food animal sector, but the exact magnitude of prevalence should be interpreted with caution.

The variation in ESBL prevalence across animal species is not simply a statistical artefact but a meaningful reflection of the distinct production systems, biosecurity standards, and antibiotic use cultures associated with each livestock category in the Nigerian context. The elevated burden in small ruminants, particularly goats, is consistent with the informal and largely unregulated nature of small ruminant husbandry in Nigeria, where animals are frequently maintained in peri-urban settings with unrestricted access to over-the-counter antimicrobials without veterinary guidance [42]. Published data from Nigeria indicate that over 77% of livestock farm owners use antibiotics without veterinary direction, primarily to promote growth and prevent disease [48,49,50], a practice that creates sustained selection pressure favoring the emergence and persistence of ESBL-encoding organisms, most notably *bla*_CTX-M-15_, the dominant gene variant in Nigerian and West African livestock [19,41]. However, ESBL-mediated resistance in livestock is not limited to *bla*_CTX-M_ alone. Other gene families, including *bla*_TEM_ and *bla*_SHV_, have also been reported, often co-existing within the same bacterial populations and carried on mobile genetic elements such as plasmids. This genetic diversity facilitates horizontal transfer of resistance determinants across bacterial species and ecological niches, reinforcing the role of livestock systems as important reservoirs for the amplification and dissemination of ESBL-associated resistance [51].

The elevated ESBL burden in cattle warrants particular attention, given their dual public health significance as sources of both meat and milk for human consumption. Cattle are well-established long-term reservoirs of ESBL-encoding conjugative plasmids, capable of sustaining resistant populations across production cycles and transmitting resistance determinants horizontally to co-resident bacterial species within the farm ecosystem [13,52]. Globally and regionally, *bla*_CTX-M-15_ has been identified as the dominant ESBL type in cattle across African countries, and its frequent association with broad-host-range IncF plasmids makes it particularly concerning from a transmission standpoint [13,16]. In avian species, which are the most extensively studied species in this review, the moderate pooled prevalence should not be interpreted as reassuring. Poultry in Nigeria, as across sub-Saharan Africa more broadly, are subject to prophylactic and metaphylactic antibiotic use as a routine substitute for biosecurity and vaccination programmes [53,54,55,56]. A systematic review of ESBL-producing *E. coli* in African poultry confirmed that phenotypic ESBL expression was detected across a wide colonisation range in sampled poultry, with methodological differences and the absence of standardized sampling strategies complicating cross-study comparison, a challenge mirrored in the present review [53].

The persistent and substantial heterogeneity within the avian and porcine subgroups in this review further suggests that ESBL prevalence in these species is highly context-dependent, shaped by farm-level antibiotic use and management practices that national-level pooled estimates inevitably obscure. The very low prevalence observed in ovine is likely a reflection of the limited number of contributing studies (n = 2) rather than a genuine biological difference in colonisation susceptibility and should be interpreted with caution. The species-level data reinforce the need for species-specific antimicrobial stewardship approaches rather than a one-size-fits-all regulatory response.

One of the most consequential findings of this review is that ESBL-producing *E. coli* were detected in animal-derived food products at prevalence levels comparable to those in live animals. This equivalence is epidemiologically significant because it confirms that resistant organisms are not being meaningfully eliminated during slaughter, processing, and distribution; a finding consistent with the report of Ribeiro [8]. In Nigeria, where informal slaughter practices, limited cold-chain infrastructure, and inadequate food hygiene enforcement are widespread, the conditions for sustaining and amplifying microbial contamination throughout the processing chain are structurally embedded in the food system [57]. The strikingly low proportion of studies examining food products relative to live animals further reveals a research blind spot because contamination rates in processed products are shaped by slaughter hygiene, cross-contamination dynamics, storage, and retail handling, none of which are captured by animal carriage studies alone. Future surveillance programmes must routinely sample retail meat, milk, and eggs at the point of sale to characterize the true scope of consumer exposure.

The regional variation in ESBL-producing *Escherichia coli* prevalence across Nigeria’s geopolitical zones likely reflects differences in agro-ecological conditions, livestock production systems, and antimicrobial use practices. The South-West, South-South and South-East, where higher prevalence was observed, host a large proportion of Nigeria’s intensive commercial livestock operations, particularly poultry and pig farming, where prophylactic and metaphylactic antibiotic use is widespread [58,59]. In addition, the humid tropical climate of southern Nigeria may favor the environmental persistence and dissemination of resistant organisms through soil, water, and manure, facilitating the amplification and horizontal transfer of ESBL-encoding mobile genetic elements within agricultural ecosystems [8,60]. However, the geographical distribution of studies included in this review was uneven, with a disproportionate representation from the South-West region of Nigeria. This likely reflects differences in research capacity, institutional presence, and laboratory infrastructure rather than true epidemiological distribution. As a result, pooled prevalence estimates may be influenced by regional sampling bias and may not fully represent the national burden of ESBL-producing *E. coli*. Increased surveillance and research efforts in underrepresented regions, particularly in northern Nigeria, are necessary to provide a more balanced and nationally representative understanding of antimicrobial resistance patterns.

The discrepancy in pooled prevalence between studies employing combined phenotypic and molecular detection methods versus those relying on phenotypic culture alone carries important methodological implications that extend beyond this review. Phenotypic confirmatory tests are dependent on enzyme expression under laboratory conditions and are known to underperform in detecting strains carrying low-expression or atypical ESBL gene variants, whereas molecular methods directly identify resistance determinants irrespective of phenotypic expression levels [61]. This methodological difference directly contributes to the higher prevalence estimates observed in studies employing combined phenotypic and molecular approaches compared to those relying on phenotypic methods alone. The implication is that surveillance programmes relying exclusively on phenotypic methods systematically underestimate the true prevalence of ESBL-carrying organisms in animal populations, a particularly consequential form of measurement bias in a setting where the documented burden already fails to generate adequate policy urgency. Beyond improving sensitivity, the wider adoption of molecular and whole-genome sequencing approaches in Nigerian veterinary AMR surveillance would enable genotypic characterisation of circulating ESBL variants, particularly the predominant *bla*_CTX-M-15_-plasmid diversity mapping, and phylogenetic analysis of shared resistance gene clusters between animal and human clinical isolates [8,16]. Such data are essential for determining whether the ESBL genes identified in Nigerian livestock are phylogenetically related to those causing clinical infections in humans, a question that existing evidence cannot answer but that is fundamental to establishing the true public health significance of the animal reservoir. Harmonization of diagnostic protocols across Nigerian veterinary and public health laboratories, anchored in internationally recognized standards, is therefore a prerequisite not only for research comparability but for the integrity of any future national AMR surveillance system.

These findings must be interpreted within their structural context as Nigeria lacks a national veterinary antimicrobial consumption monitoring system, critically important antibiotics remain widely available over the counter without prescription [62,63,64], and the country’s first National Action Plan on AMR (2017–2022) achieved only a 44% completion rate with limited agricultural sector engagement [65]. While the launch of NAP 2.0 (2024–2028) represents a positive step, its impact will depend on addressing the persistent implementation gaps that have allowed resistance to accumulate unchecked in the livestock sector [65].

From a One Health perspective, the significance of these findings extends well beyond veterinary medicine. ESBL-producing *E. coli* are paradigmatic pathogens at the animal–human–environment interface: resistant strains circulating in livestock enter the environment through manure and wastewater, contaminate food products through slaughter and processing, and reach human populations through both foodborne exposure and direct contact during animal handling [8,13]. In Nigeria, where a large proportion of the population depends on locally produced animal protein and occupational exposure among livestock farmers, slaughterhouse workers, and veterinary personnel is extensive and largely unmonitored, the pathway from animal reservoir to human infection is structurally embedded in everyday life [16].

Clinically, ESBL-producing *E. coli* infections are associated with treatment failure, prolonged hospitalization, and excess mortality; outcomes amplified in settings with limited carbapenem access and inadequate infection control infrastructure [61,66]. ESBL dissemination through the food chain therefore represents an active contributor to Nigeria’s contemporary clinical AMR burden, disproportionately affecting populations least able to withstand it. The urgency of intervention is underscored by global modelling projections that antibiotic use in Nigerian food animals will increase by an estimated 163% by 2030 under a business-as-usual scenario [67], a trajectory incompatible with any meaningful containment of ESBL resistance. Coordinated One Health action, integrating veterinary prescription enforcement, national antimicrobial use monitoring, and investment in biosecurity and vaccination as alternatives to prophylactic antibiotic use, is essential to reverse this trajectory.

## 4. Materials and Methods

### 4.1. Study Design

The present investigation was designed as a systematic review and meta-analysis aimed at estimating the pooled prevalence of extended-spectrum beta-lactamase (ESBL)-producing *Escherichia coli* in food-producing animals and animal-derived food products within Nigeria. The review was conceptualized and reported in strict adherence to the Preferred Reporting Items for Systematic Reviews and Meta-Analyses (PRISMA) guidelines (Supplementary S1).

The conduct of this systematic review was guided by a protocol prospectively registered in PROSPERO (registration number: CRD420250650516). No substantive deviations from the registered protocol were encountered throughout the review process. Minor operational refinements encompassing clarification of subgroup definitions and the broadening of sensitivity analyses were introduced during the course of the review to enhance methodological rigor and address recommendations arising from peer-review feedback, without modification of the original research questions or eligibility criteria. Studies were considered eligible for inclusion if they were cross-sectional in design, conducted within Nigeria, reported primary data on the occurrence of ESBL-producing *E. coli* in food-producing animals (including poultry, cattle, pigs, goats, and sheep) and/or animal-derived food products (including meat, milk, and eggs), and employed phenotypic and/or molecular methods for ESBL detection and confirmation.

### 4.2. Data Sources and Search Strategy

In accordance with PRISMA recommendations, a rigorous and comprehensive literature search was undertaken across four electronic databases: PubMed, Web of Science, Scopus, and African Journals Online (AJOL). The search strategy was constructed using combinations of the following keywords and Boolean operators: “*Escherichia coli*” OR “*E. coli*” AND “extended-spectrum beta-lactamase” OR “ESBL” OR “ESBL-producing” AND “food-producing animals” OR livestock OR poultry OR cattle OR pigs OR goats OR sheep OR “animal-derived food products” OR meat OR milk OR eggs AND Nigeria (Supplementary S2). The search encompassed literature published between January 2000 and January 2026, with the final search executed on 31 January 2026. Eligibility was restricted to studies published in the English language, owing to resource limitations and the imperative for accurate interpretation of study findings. Furthermore, searches were confined to peer-reviewed journal articles. Grey literature, conference abstracts, theses, and preprints were deliberately excluded to maintain consistency in methodological quality, ensure the availability of comprehensive study details, and uphold the reliability of diagnostic reporting. Although this approach may constrain the retrieval of unpublished data, it substantially mitigates the risk of incorporating studies that have not undergone formal peer review. The complete search strings for each database are detailed in the Supplementary Material (Supplementary S2). Additionally, reference lists of all included articles and pertinent reviews were manually scrutinized to identify any further eligible studies not captured through the electronic database searches. ESBL-producing *E. coli* were defined as isolates exhibiting resistance to third-generation cephalosporins and confirmed using phenotypic confirmatory tests (double-disk synergy test or combination disk methods), with or without molecular confirmation of ESBL-associated genes. Studies using recognized laboratory standards, including CLSI or equivalent protocols, were considered eligible.

### 4.3. Selection and Extraction of Data from Included Studies

All retrieved citations were imported into EndNote 21 (Clarivate Analytics) for systematic reference management. Initial deduplication was performed utilizing the software’s integrated “Find Duplicates” function, subsequently supplemented by manual verification to ensure the thoroughness and completeness of duplicate removal. The remaining unique records were subsequently exported into Microsoft Excel, whereupon two independent reviewers conducted parallel screening of titles and abstracts against a standardized eligibility form. Full-text assessment and data extraction were likewise performed independently by the same two reviewers. Discrepancies arising between reviewers at any stage of the screening or extraction process were resolved through structured discussion and mutual consensus; in instances where agreement could not be achieved, adjudication was sought from a third reviewer to ensure the integrity and objectivity of the selection process. Extracted data included: Author(s) and year of publication, study location (state and geopolitical region), animal species or food product sampled, sample size (total number tested), number of ESBL-positive *E. coli* isolates, sample type, ESBL detection method (phenotypic culture-based methods alone or combined phenotypic and molecular methods).

### 4.4. Inclusion and Exclusion Criteria

#### 4.4.1. Inclusion Criteria

Studies were eligible if they:

1. Reported original data on the occurrence of ESBL-producing *E. coli*.
2. Involved food-producing animals (e.g., poultry, cattle, pigs, goats, sheep) and/or animal-derived food products (e.g., meat, milk, eggs).
3. Were conducted in Nigeria.
4. Employed phenotypic and/or molecular methods for ESBL detection.
5. Provided sufficient data to extract or calculate prevalence estimates (numerator and denominator).

#### 4.4.2. Exclusion Criteria

Studies were excluded if:

1. Conducted in geographical settings outside Nigeria.
2. Did not report quantitative data pertaining to ESBL-producing *E. coli.*
3. Classified as review articles, commentaries, editorials, or outbreak notifications devoid of original empirical data.
4. Full-text versions were unavailable or lacked sufficient methodological detail to permit rigorous quality assessment.

### 4.5. Study Quality Assessment

The methodological quality of all included studies was appraised utilizing the Joanna Briggs Institute (JBI) critical appraisal checklists appropriate to the respective study designs. To facilitate consistent interpretation and comparability of quality assessments across studies, scores were categorized according to percentage-based thresholds congruent with those adopted in previous systematic reviews employing JBI appraisal instruments. Studies fulfilling ≥70% of applicable appraisal items were designated as high quality, those satisfying 50–69% were classified as moderate quality, and those meeting fewer than 50% of applicable criteria were categorized as low quality. These thresholds were deliberately selected to discriminate between studies exhibiting minimal risk of bias and those bearing substantial methodological limitations, while simultaneously precluding the application of unduly restrictive exclusion criteria in a field characterized by a paucity of systematic surveillance data. Analogous cut-off approaches have been previously adopted in meta-analyses of zoonotic and infectious diseases as a means of balancing methodological stringency with the need for inclusiveness (Supplementary S3).

### 4.6. Data Analysis

Extracted data were systematically organized in Microsoft Excel and subsequently analyzed using OpenMeta (Analyst, version 0.30.0) and Comprehensive Meta-Analysis (CMA) version 3.0 software. Pooled prevalence estimates were derived through the application of random-effects meta-analysis models, selected to appropriately accommodate the anticipated magnitude of between-study heterogeneity. Between-study variance was estimated using the DerSimonian–Laird estimator, consistent with methodological approaches widely adopted in meta-analyses of antimicrobial resistance prevalence and to ensure comparability with the existing literature.

Prior to analysis, all proportions underwent logit transformation to stabilize variances and were subsequently back-transformed to facilitate meaningful interpretation of results. Between-study heterogeneity was evaluated using Cochran’s Q test, with statistical significance defined at *p* < 0.05, and further quantified through the I^2^ statistic, whereby values ≥ 75% were interpreted as indicative of substantial heterogeneity. Predefined subgroup analyses were executed to systematically investigate potential sources of heterogeneity across the following strata: animal species, animal-derived food products, sample type, ESBL detection methodology (phenotypic methods exclusively versus combined phenotypic and molecular approaches), geopolitical region, and state. Studies contributing data to multiple subgroups were incorporated into all relevant subgroup analyses; however, each study was enumerated only once in the computation of the overall pooled estimate. Given the persistence of considerable heterogeneity across subgroups, findings were interpreted descriptively. Sensitivity analyses were undertaken to interrogate the robustness of the pooled estimates by systematically evaluating the influence of individual studies and those reporting extreme prevalence values. No single study was identified as exerting a disproportionate influence on the overall pooled prevalence estimate.

Assessment of publication bias and small-study effects pertaining to the overall pooled prevalence was conducted through funnel plot visualization and Egger’s regression test. In light of the substantial heterogeneity observed, the findings of these assessments were interpreted with due caution, acknowledging that funnel plot asymmetry may be attributable to genuine study-level differences rather than selective publication practices. All statistical tests were two-tailed, with a significance threshold of *p* < 0.05. Meta-analytic results are presented in the form of forest plots accompanied by corresponding confidence intervals and study weights.

## 5. Conclusions

This systematic review and meta-analysis demonstrates a substantial prevalence of ESBL-producing *Escherichia coli* across food-producing animals and animal-derived food products in Nigeria, with variation by species, geography, and diagnostic approach. The findings identify the livestock sector as an important reservoir of antimicrobial resistance determinants and highlight critical gaps in surveillance, including limited geographic coverage, insufficient monitoring of food products, and poor integration of animal and human AMR data. Addressing these gaps requires a coordinated One Health approach that strengthens antimicrobial stewardship, food safety systems, and integrated surveillance.

## 6. Limitation

The included studies exhibited substantial heterogeneity in sampling strategies, laboratory methods, study populations, and geographical coverage, which may affect comparability and the precision of pooled prevalence estimates. In addition, the uneven geographical distribution of studies, with limited representation from several Nigerian geopolitical zones, restricts the generalizability of national-level inferences. Differences in diagnostic methodologies, particularly between phenotypic and molecular approaches, may have further contributed to variability in reported prevalence across studies. Although the included studies span over two decades (2000–2025), a formal temporal trend analysis was not conducted due to the uneven distribution of studies across time periods and the limited number of studies within specific intervals, which may reduce the reliability of such analyses. Consequently, changes in ESBL prevalence over time could not be robustly assessed.

Furthermore, the restriction of included studies to English-language publications may have introduced selection bias, as relevant studies published in other languages may have been excluded. Although English is the predominant language of scientific publication in Nigeria, this limitation should be considered when interpreting the findings.

## Data Availability

The data sets used and/or analyzed during the current study are available within the manuscript and supplementary file.

## Supplementary Materials

The following supporting information can be downloaded at: https://www.mdpi.com/article/10.3390/antibiotics15050432/s1, Supplementary S1: PRISMA Checklist; Supplementary S2: Detailed Search Strategy for ESBL-Producing *Escherichia coli* in Nigeria; Supplementary S3: Methodological quality assessment of included studies using the Joanna Briggs Institute (JBI) checklist for prevalence studies.

## Abbreviations

The following abbreviations are used in this manuscript:

AMR	Antimicrobial Resistance
AJOL	African Journals Online
*bla*_CTX-M_	Beta-Lactamase Gene (Cefotaxime-Munich Type)
*bla*_CTX-M-15_	Beta-Lactamase CTX-M Group 15 Variant
*bla*_TEM_	Beta-Lactamase TEM-Type Gene
CI	Confidence Interval
CLSI	Clinical and Laboratory Standards Institute
CMA	Comprehensive Meta-Analysis
*E. coli*	*Escherichia coli*
ESBL	Extended-Spectrum Beta-Lactamase
FCT	Federal Capital Territory
I^2^	Inconsistency Statistic (Measure of Between-Study Heterogeneity)
IncF	Incompatibility Group F Plasmid
JBI	Joanna Briggs Institute
LMICs	Low- and Middle-Income Countries
MRSA	Methicillin-Resistant Staphylococcus aureus
NAFDAC	National Agency for Food and Drug Administration and Control
NAP	National Action Plan (on AMR)
PRISMA	Preferred Reporting Items for Systematic Reviews and Meta-Analyses
PROSPERO	International Prospective Register of Systematic Reviews
SSA	Sub-Saharan Africa
WHO	World Health Organization
95% CI	95% Confidence Interval
K	Number of Contributing Datasets (in Meta-Analysis)
N	Sample Size/Number of Studies
*P*	*p*-value (Statistical Significance)
T	Egger’s Regression Test Statistic
