# Supplementary material for: Systematic Review and Meta-Analysis of Extended-Spectrum Beta-Lactamase-Producing (ESBL) Escherichia coli in Food-Producing Animals and Animal Products in Nigeria
Source: Antibiotics (Basel). 2026 Apr 25;15(5):432. doi: 10.3390/antibiotics15050432 (PMC13203192; doi:10.3390/antibiotics15050432)
Supplement: Supplementary file 1 [file antibiotics-15-00432-s001.zip › antibiotics-4237983-supplementary.pdf]

Supplementary S1: PRISMA Checklist

Section and Topic	Item #	Checklist item	Location where item is reported
**TITLE**			
Title	1	Identify the report as a systematic review.	1
**ABSTRACT**			
Abstract	2	Abstract	1
**INTRODUCTION**			
Rationale	3	Describe the rationale for the review in the context of existing knowledge.	4
Objectives	4	Provide an explicit statement of the objective(s) or question(s) the review addresses.	5
**METHODS**			
Eligibility criteria	5	Specify the inclusion and exclusion criteria for the review and how studies were grouped for the syntheses.	7
Information sources	6	Specify all databases, registers, websites, organizations, reference lists, and other sources searched or consulted to identify studies. Specify the date when each source was last searched or consulted.	6
Search strategy	7	Present the complete search strategies for all databases, registers, and websites, including any filters and limits used.	Suppl. 2
Selection process	8	Specify the methods used to decide whether a study met the review's inclusion criteria, including how many reviewers screened each record and each report retrieved, whether they worked independently, and, if applicable, details of automation tools used in the process.	8
Data collection process	9	Specify the methods used to collect data from reports, including how many reviewers collected data from each report, whether they worked independently, any processes for obtaining or confirming data from study investigators, and, if applicable, details of automation tools used in the process.	8
Data items	10a	List and define all outcomes for which data were sought. Specify whether all results that were compatible with each outcome domain in each study were sought (e.g., for all measures, time points, analyses), and if not, the methods used to decide which results to collect.	8
	10b	List and define all other variables for which data were sought (e.g., participant and intervention characteristics, funding sources). Describe any assumptions made about any missing or unclear information.	7
Study quality assessment	11	Specify the methods used to assess the quality of the included studies, including details of the tool(s) used, how many reviewers assessed each study and whether they worked independently, and if applicable, details of automation tools used in the process.	8, Suppl. 3
Effect measures	12	Specify for each outcome the effect measure(s) (e.g., risk ratio, mean difference) used in the synthesis or presentation of results.	n/a as narrative synthesis
Synthesis methods	13a	Describe the processes used to decide which studies were eligible for each synthesis (e.g., tabulating the study intervention characteristics and comparing against the planned groups for each synthesis (item #5)).	10

Section and Topic	Item #	Checklist item	Location where item is reported
	13b	Describe any methods required to prepare the data for presentation or synthesis, such as handling missing summary statistics or data conversions.	9
	13c	Describe any methods used to tabulate or visually display the results of individual studies and syntheses.	9
	13d	Describe any methods used to synthesize results and provide a rationale for the choice(s). If meta-analysis was performed, describe the model(s), method(s) to identify the presence and extent of statistical heterogeneity, and software package(s) used.	9
	13e	Describe any methods used to explore possible causes of heterogeneity among study results (e.g., subgroup analysis, meta-regression).	9
	13f	Describe any sensitivity analyses conducted to assess the robustness of the synthesized results.	9
Reporting bias assessment	14	Describe any methods used to assess the risk of bias due to missing results in a synthesis (arising from reporting biases).	9
Certainty assessment	15	Describe any methods used to assess certainty (or confidence) in the body of evidence for an outcome.	9
**RESULTS**			
Study selection	16a	Describe the results of the search and selection process, from the number of records identified in the search to the number of studies included in the review, ideally using a flow diagram.	10
Study characteristics	17	Cite each included study and present its characteristics.	10, Table 1
Quality assessment of studies	18	Present assessments of quality for each included study.	Suppl. 3
Results of individual studies	19	For all outcomes, present, for each study: (a) summary statistics for each group (where appropriate) and (b) an effect estimate and its precision (e.g., confidence/credible interval), ideally using structured tables or plots.	10-15
Results of syntheses	20a	For each synthesis, briefly summarise the characteristics and risk of bias among contributing studies.	Suppl. 3
	20b	Present results of all statistical syntheses conducted. If meta-analysis was done, present for each the summary estimate and its precision (e.g., confidence/credible interval) and measures of statistical heterogeneity. If comparing groups, describe the direction of the effect.	10-15
	20c	Present results of all investigations of possible causes of heterogeneity among study results.	10-15
	20d	Present results of all sensitivity analyses conducted to assess the robustness of the synthesized results.	10
Reporting biases	21	Present assessments of risk of bias due to missing results (arising from reporting biases) for each synthesis assessed.	12, Fig 2

Section and Topic	Item #	Checklist item	Location where item is reported
Certainty of evidence	22	Present assessments of certainty (or confidence) in the body of evidence for each outcome assessed.	8
**DISCUSSION**			
Discussion	23a	Provide a general interpretation of the results in the context of other evidence.	15-22
	23b	Discuss any limitations of the evidence included in the review.	22
	23c	Discuss any limitations of the review processes used.	n/a
	23d	Discuss implications of the results for practice, policy, and future research.	22
**OTHER INFORMATION**			
Registration and protocol	24a	Provide registration information for the review, including register name and registration number, or state that the review was not registered.	6
	24b	Indicate where the review protocol can be accessed or state that a protocol was not prepared.	6
	24c	Describe and explain any amendments to the information provided at registration or in the protocol.	n/a
Support	25	Describe sources of financial or non-financial support for the review and the role of the funders or sponsors in the review.	23
Competing interests	26	Declare any competing interests of review authors.	23
Availability of data, code, and other materials	27	Report which of the following are publicly available and where they can be found: template data collection forms; data extracted from included studies; data used for all analyses; analytic code; any other materials used in the review.	All relevant data are within the manuscript and supplementary file

(Source: Page MJ et al., 2021 [24] or http://www.prisma-statement.org/ (accessed on 15 January 2026).

Supplementary S2: Detailed Search Strategy for ESBL-Producing *Escherichia coli* in Nigeria

A comprehensive and systematic literature search was conducted in four databases: PubMed, African Journals Online (AJOL), Scopus, and Web of Science (Core Collection) to identify peer-reviewed studies reporting on the prevalence and occurrence of extended-spectrum beta-lactamase (ESBL)-producing *Escherichia coli* in food-producing animals and animal-derived food products in Nigeria.

The search covered publications from January 2000 to January 2026, corresponding to the early emergence and subsequent reporting of ESBL-producing Enterobacterales in Nigeria, and was restricted to studies published in English.

PubMed

The PubMed search strategy combined Medical Subject Headings (MeSH) and free-text keywords to maximize coverage and sensitivity. The final search string used was:

("Escherichia coli"[MeSH Terms] OR "Escherichia coli"[Title/Abstract] OR "E. coli"[Title/Abstract])

AND

("beta-Lactamases"[MeSH Terms] OR "extended-spectrum beta-lactamase"[Title/Abstract] OR "ESBL"[Title/Abstract] OR "ESBL-producing"[Title/Abstract])

AND

("Animals, Food-Producing"[MeSH Terms] OR livestock[Title/Abstract] OR poultry[Title/Abstract]

OR cattle[Title/Abstract] OR cow*[Title/Abstract] OR pig*[Title/Abstract]

OR goat*[Title/Abstract] OR sheep[Title/Abstract]

OR "animal-derived food products"[Title/Abstract]

OR meat[Title/Abstract] OR milk[Title/Abstract] OR egg*[Title/Abstract])

AND

("Nigeria"[Title/Abstract])

Filters applied:

- Language: English
- Species: Animals
- Article type: Original research articles
- Publication years: 2000-2026

Records identified from PubMed: 61

Scopus

In Scopus, searches were conducted using the TITLE-ABS-KEY fields to capture occurrences in titles, abstracts, or keywords:

TITLE-ABS-KEY ("Escherichia coli" OR "E. coli")

AND

TITLE-ABS-KEY ("extended-spectrum beta-lactamase" OR ESBL OR "ESBL-producing")

AND

TITLE-ABS-KEY (livestock OR poultry OR cattle OR pigs OR goats OR sheep

OR "food-producing animals" OR meat OR milk OR eggs)

AND

TITLE-ABS-KEY (Nigeria)

Filters applied:

- Document type: Article
- Subject area: Veterinary Science, Microbiology, Epidemiology, Food Safety, Public Health
- Language: English

Records identified from Scopus: 53

Web of Science (Core Collection)

In Web of Science, searches were performed using the TS= (Topic) field, which includes title, abstract, author keywords, and Keywords Plus®:

TS=("Escherichia coli" OR "E. coli")

AND

TS=("extended-spectrum beta-lactamase" OR ESBL OR "ESBL-producing")

AND

TS=("food-producing animals" OR livestock OR poultry OR cattle OR pigs

OR goats OR sheep OR meat OR milk OR eggs)

AND

TS=(Nigeria)

Filters applied:

- Document type: Articles and Reviews
- Language: English
- Publication years: 2000-2026

Records identified from Web of Science: 39

African Journals Online (AJOL)

Given the limited Boolean search capabilities of AJOL, simple search combinations were used sequentially. Each search was run independently, and retrieved records were manually screened for relevance.

Examples of search strings:

- "ESBL" AND "*Escherichia coli*" AND "Nigeria"
- "extended-spectrum beta-lactamase" AND "livestock"
- "ESBL" AND "poultry" AND "Nigeria"
- "ESBL-producing *E. coli*" AND "meat"
- "ESBL" AND "milk" AND "Nigeria"
- "antimicrobial resistance" AND "*Escherichia coli*" AND "animals"

Records identified from AJOL: 19

Additional Manual and Reference Searches

- Reference lists of all included articles were manually screened to identify additional eligible studies not captured by database searches.
- Relevant review articles were also screened for potentially eligible primary studies.

Supplementary S3: Methodological quality assessment of included studies using the Joanna Briggs Institute (JBI) checklist for prevalence studies

Study	Study Design	State	Method of Detection	Q1	Q2	Q3	Q4	Q5	Q6	Q7	Q8	Q9	Score	%	Quality
Adefioye et al., 2021a	Cross-sectional	Oyo and Osun	Culture and Molecular	1	1	1	1	1	1	1	1	1	9	100	High
Adefioye et al., 2021b	Cross-sectional	Oyo and Osun	Culture and Molecular	1	1	1	1	1	1	1	1	1	9	100	High
Adefioye et al., 2021c	Cross-sectional	Oyo and Osun	Culture and Molecular	1	1	1	1	1	1	1	1	1	9	100	High
Adefioye et al., 2021d	Cross-sectional	Oyo and Osun	Culture and Molecular	1	1	1	1	1	1	1	1	1	9	100	High
Adefioye et al., 2021e	Cross-sectional	Oyo and Osun	Culture and Molecular	1	1	1	1	1	1	1	1	1	9	100	High
Adefioye et al., 2021f	Cross-sectional	Oyo and Osun	Culture and Molecular	1	1	1	1	1	1	1	1	1	9	100	High
Adenipekun et al., 2015a	Cross-sectional	Lagos	Culture and Molecular	1	1	1	1	1	1	1	1	1	9	100	High
Adenipekun et al., 2015b	Cross-sectional	Lagos	Culture and Molecular	1	1	1	1	1	1	1	1	1	9	100	High
Adenipekun et al., 2015c	Cross-sectional	Lagos	Culture and Molecular	1	1	1	1	1	1	1	1	1	9	100	High
Adetunji et al., 2025	Cross-sectional	Oyo	Culture and Molecular	1	1	1	1	1	1	1	1	1	9	100	High

Al-Mustapha et al., 2023	Cross-sectional	Kwara	Culture and Molecular	1	1	1	1	1	1	1	1	1	9	100	High
Anueyiagu et al., 2022	Cross-sectional	Plateau	Culture and Molecular	1	1	1	1	1	1	1	1	1	9	100	High
Aworh et al., 2020	Cross-sectional	FCT	Culture and Molecular	1	1	1	1	1	1	1	1	1	9	100	High
Aworh et al., 2022	Cross-sectional	Lagos and FCT	Culture and Molecular	1	1	1	1	1	1	1	1	1	9	100	High
Chika et al., 2018	Cross-sectional	Ebonyi	Culture and Molecular	1	1	1	1	1	1	1	1	1	9	100	High
Fashae et al., 2021a	Cross-sectional	Oyo	Culture and Molecular	1	1	1	1	1	1	1	1	1	9	100	High
Fashae et al., 2021b	Cross-sectional	Oyo	Culture and Molecular	1	1	1	1	1	1	1	1	1	9	100	High
Fortini et al., 2011a	Cross-sectional	Oyo	Culture and Molecular	1	1	1	1	1	1	1	1	1	9	100	High
Fortini et al., 2011b	Cross-sectional	Oyo	Culture and Molecular	1	1	1	1	1	1	1	1	1	9	100	High
Kabir et al., 2025	Cross-sectional	Sokoto	Culture and Molecular	1	1	1	1	1	1	1	1	1	9	100	High
Nnaji et al., 2021	Cross-sectional	Enugu and Ebonyi	Culture and Molecular	1	1	1	1	1	1	1	1	1	9	100	High
Olowe et al., 2015a	Cross-sectional	Ekiti	Culture and Molecular	1	1	1	1	1	1	1	1	1	9	100	High

Olowe et al., 2015b	Cross-sectional	Ekiti	Culture and Molecular	1	1	1	1	1	1	1	1	1	9	100	High
Nsofor et al., 2018	Cross-sectional	Imo	Culture and Molecular	1	1	1	1	1	1	1	1	1	9	100	High
Amosun et al., 2024a	Cross-sectional	Oyo	Culture	1	1	1	1	1	1	1	0	0	7	77.8	Moderate
Amosun et al., 2024b	Cross-sectional	Oyo	Culture	1	1	1	1	1	1	1	0	0	7	77.8	Moderate
Amosun et al., 2024c	Cross-sectional	Oyo	Culture	1	1	1	1	1	1	1	0	0	7	77.8	Moderate
Kwoji et al., 2019	Cross-sectional	Borno	Culture	1	1	1	1	1	1	1	0	0	7	77.8	Moderate
Lawan et al., 2024a	Cross-sectional	Borno	Culture	1	1	1	1	0	0	0	0	0	4	44.4	Low
Lawan et al., 2024b	Cross-sectional	Borno	Culture	1	1	1	1	0	0	0	0	0	4	44.4	Low
Olopade et al., 2022	Cross-sectional	Plateau	Culture	1	1	1	1	0	0	0	0	0	4	44.4	Low
Egbule and Yusuf, 2019a	Cross-sectional	Delta	Culture	1	1	1	1	0	0	0	0	0	4	44.4	Low
Egbule and Yusuf, 2019b	Cross-sectional	Delta	Culture	1	1	1	1	0	0	0	0	0	4	44.4	Low
Ugwu et al., 2018	Cross-sectional	Anambra	Culture	1	1	1	1	0	0	0	0	0	4	44.4	Low

Scoring: Yes = 1; No/Unclear = 0, Quality classification: High: ≥70%, Moderate: 50-69%, Low: <50%

JBI critical appraisal checklist for prevalence studies

Q1. Appropriate sample frame

Q2. Appropriate sampling method

Q3. Adequate sample size

Q4. Detailed description of subjects and setting

Q5. Data analysis with sufficient coverage

Q6. Valid methods for identifying ESBL-producing *E. coli*

Q7. Reliable measurement of the condition

Q8. Appropriate statistical analysis

Q9. Adequate response rate
